# Unveiling the Potential of *Lentilactobacillus hilgardii* in Malolactic Fermentation: Comparative Genomics and Fermentation Dynamics

**DOI:** 10.1111/1751-7915.70259

**Published:** 2025-11-30

**Authors:** Giacomo Mantegazza, Nicola Mangieri, Elnaz Vojoudi Yazdi, Pasquale Russo, Diego Mora, Giorgio Gargari

**Affiliations:** ^1^ Laboratory of Food Systems Biotechnology Department of Health Sciences and Technology, ETH Zurich Switzerland; ^2^ Department of Food, Environmental and Nutritional Sciences (DeFENS) University of Milan Milan Italy

**Keywords:** biogenic amines, genome comparison, genome‐scale metabolic models, *Lentilactobacillus hilgardii*, malolactic fermentation, *Oenococcus oeni*, pangenome, potential metabolic prediction

## Abstract

This study aimed to assess the potential of *Lentilactobacillus hilgardii* as a novel candidate for malolactic fermentation (MLF) in winemaking, through comparative genomics and experimental validation, in direct comparison with *Oenococcus oeni* . We performed a pangenome analysis on 16 *L. hilgardii* and 7 *O. oeni* strains to explore their genetic diversity, focusing on wine‐related traits. Functional predictions were generated using genome‐scale metabolic models (ModelSEED/KBase), including in silico co‐inoculation with *Saccharomyces cerevisiae* EC1118 and post‐alcoholic fermentation simulations. The reference strains *L. hilgardii* DSM 20176 and *O. oeni* DSM 20252 were experimentally tested for MLF performance in a synthetic wine‐like medium at 25°C and 10°C. Core‐genome comparison revealed that 67.9% of the malolactic enzyme sequence is conserved between the two species, with comparable docking affinity to L‐malic acid. *L. hilgardii* harboured unique enzymes with potential oenological interest (phenolic acid decarboxylase, mannitol dehydrogenase, glucosidase) and distinctive stress‐related proteins (YaaA, HrcA, ASP23), suggesting improved tolerance to oxidative, temperature, and alkaline stresses. Notably, *L. hilgardii* showed genomic potential to degrade putrescine, arginine, and ornithine, precursors of ethyl carbamate. Experimentally, *L. hilgardii* reduced L‐malic acid from 2.5 g/L to < 0.1 g/L within 12 days at 10°C, while *O. oeni* showed no MLF activity at this temperature. At 25°C, both strains completed MLF within 6–7 days. *L. hilgardii* also consumed > 80% of residual fructose at 10°C, whereas *O. oeni* showed minimal utilisation. Our results demonstrate that *L. hilgardii* combines a favourable genomic repertoire for wine adaptation with superior MLF performance at low temperature, suggesting its potential as an alternative to *O. oeni* in cool‐climate winemaking. This work provides the first genome‐scale comparative and functional evaluation of *L. hilgardii* in the winemaking context, highlighting its technological promise to improve fermentation reliability, reduce spoilage risk, and expand the biodiversity of malolactic starters.

## Graphical abstract

This study explores the metabolic potential of *Lentilactobacillus hilgardii* for malolactic fermentation in winemaking. Genomic and in silico analyses, combined with experimental validation, reveal its resilience at low temperatures and unique enzymatic traits. Comparative insights with *Oenococcus oeni* highlight *L. hilgardii* as a promising alternative malolactic starter under challenging wine conditions.

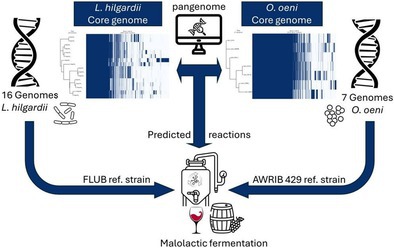

**Abbreviations**
FAN: free amino nitrogen
LAB: lactic acid bacteria
MLF: malolactic fermentation

## 1 Introduction

Comparative genomics using pangenome analysis is a powerful approach for studying the genetic diversity within a species or across multiple related species (Hyun et al. 2022). The pangenome refers to the entire set of genes present in a group of organisms, consisting of the core genome (genes shared by all members) and the accessory genome (genes present in some but not all members). Through this comparative genomics approach, it is possible to gain insights into the genetic basis of wine‐related traits, which can be valuable for improving winemaking processes, ensuring product consistency, and detecting strains with desirable characteristics (Borneman et al. 2016). The dearth of information regarding the genetic potential of *Lentilactobacillus hilgardii* in existing literature has underscored the necessity for an in‐depth exploration. In this study, our primary emphasis is on conducting a thorough comparative genomics analysis between two distinct species within the context of malolactic fermentation (MLF) referred to in this manuscript also as secondary fermentation.

MLF is undertaken in the production of red wines and some white wines, characterised by the bioconversion of L‐malic acid into L‐lactic acid, mediated by some lactic acid bacteria (LAB). Due to their metabolism malolactic bacteria are responsible for a series of biochemical reactions that significantly contribute to the aroma, stability and structure of the final product (Virdis et al. 2021). Moreover, they can influence the colour of the wine by enhancing the chromatic stability, a phenomenon attributed to the increased concentration of acetaldehyde and the catalytic action of glycosidases (Liu and Pilone 2000) that have the potential to hydrolyze the glycosidic bond of anthocyanins (Devi et al. 2020).

Simultaneously, proteolytic activity might contribute to the stabilisation of the wine (Sandri et al. 2011), preventing the formation of protein hazes, and a pectinolytic action that reduces the possibility of product cloudiness (Virdis et al. 2021). It should be noted that malolactic bacteria can also lead to the cleavage of the bond between acetaldehyde and sulfite, resulting in an increase in the amount of free sulfur dioxide, thus reducing the need for the addition of this additive during bottling (Virdis et al. 2021).

A significant contribution to the complexity of wine is provided by malolactic bacteria, releasing volatile compounds that can impart distinctive aromatic notes (Cappello et al. 2017). MLF in winemaking is mainly performed by *Oenococcus oeni* , a well‐adapted LAB to the harsh wine environment, that is, tolerance to high alcohol concentrations, low pH and nutritional deficiency (Mills et al. 2005; Maitre et al. 2014). In the last years, different wine‐related *Lactobacillus* spp. have been proposed as suitable MLF starter cultures (du Toit et al. 2011). In particular, *Lactiplantibacillus plantarum* strains have been extensively investigated for their technological attributes, as an alternative to *O. oeni* (Russo et al. 2020; Hu et al. 2024).

However, other less‐known malolactic bacteria, including *Lentilactobacillus hilgardii*, could be further exploited in winemaking (Rodas et al. 2003). In particular, *L. hilgardii* displays a good prevalence in Patagonian wine and a higher effectiveness in malic acid consumption than *L. plantarum* at low temperatures (4°C–10°C), suggesting its employment for vinification in cold geographic areas (Manera et al. 2023) or a superior suitability for specific winemaking techniques, such as cryomaceration and cold fermentation (Lasik 2013).

A critical concern for the selection of malolactic starter is based on the safety assessment of promising candidates. Metabolites of microbial origin with a negative impact on human health mainly include ethyl carbamate (Benito 2019), and biogenic amines (Russo et al. 2016).

A study conducted by Mazzoli and colleagues revealed the presence of histidine decarbosylase in *L. hilgardii* , responsible for the production of histamine from histidine (Mazzoli et al. 2009). Moreover, putrescine production via agmatine deaminase has been reported in some strains of *L. hilgardii* , while *L. hilgardii* IOEB 9649 was found to produce tyramine and phenylethylamine simultaneously (Moreno‐Arribas et al. 2000; Alberto et al. 2007). However, this peculiarity was strain‐specificand does not represent the entire *L. hilgardii* species (Costantini et al. 2006). On the other hand, it is noteworthy that *L. hilgardii* has the capacity to degrade biogenic amines, a genetically relevant feature that could enhance the safety of the wine (Li and Lu 2020).

In this study, we aim to explore, for the first time, the enzymatic potential of *L. hilgardii* through pangenome analysis, with the objective of evaluating its suitability as a candidate for MLF. By comparing metabolic predictions with *Oenococcus oeni* , we will identify the distinctive characteristics of the species and the reference strain *L. hilgardii* FLUB, thoroughly analysing the positive and negative aspects of its application in winemaking.

To our knowledge, this is the first study to provide an integrated genomic and metabolic comparison between *Lentilactobacillus hilgardii* and *Oenococcus oeni* with a specific focus on malolactic fermentation. Previous works have mainly concentrated on *O. oeni* as the canonical malolactic starter, or on *Lactiplantibacillus plantarum* as a versatile alternative, but no comprehensive evaluation of *L. hilgardii* has been carried out in the context of winemaking. Our study combines pangenomic analysis, metabolic modelling and experimental validation to investigate the potential of *L. hilgardii* as a novel MLF candidate, thus introducing an unexplored species into the enological context and expanding the biodiversity of malolactic starters.

## 2 Materials and Methods

### 2.1 Pangenome Analyses and Comparative Analyses

Pangenome analyses were conducted utilising *Lentolactibacillus hilgardii* strains (hilgardii_ATCC27305, hilgardii_DSM20176, hilgardii_ATCC8290, hilgardii_LH500, hilgardii_FLUB, hilgardii_LMG07934, hilgardii_TMW12196, hilgardii_TMW1828, hilgardii_Kef‐w9, hilgardii_Kef‐w10, hilgardii_Kef‐w8, hilgardii_CIRM‐BIA2119, hilgardii_CIRM‐BIA2123, hilgardii_CIRM‐BIA2118, hilgardii_60TS‐2, hilgardii_MGYG‐HGUT‐01333) and *Oenococcus oeni* strains (oeni_PSU‐1, oeni_UBOCC‐A‐315001, oeni_19, oeni_SD‐2a, oeni_AWRIB429, oeni_K19‐3, oeni_CRBO_1381) obtained from the strains already sequenced in the NCBI database. Initially, Prokka (Seemann 2014) was employed to perform genome annotation, encompassing details such as gene information, location, strandedness and various features and attributes. This comprehensive information was then stored in the GFF output file, which is a prerequisite for the subsequent step. The subsequent step involved the utilisation of Roary software (Page et al. 2015), which facilitated the generation of a phylogeny reflecting the evolutionary history of the isolates, the compilation of conserved genes, and, consequently, the construction of the pangenome.

The pangenome analyses yielded lists of cloud, shell, soft‐core and core genes. Subsequently, only the sequences identified as part of the core genes were extracted, and using these sequences, core genomes were constructed for both *L. hilgardii* and *O. oeni* species.

A comparative analysis of the core genomes was conducted manually, scrutinising the proteins that exhibited differential or similar production within each genome and focusing on the enzymes with oenological importance. The output file generated a list of genes characteristic of the core genome of the two species under consideration.

### 2.2 Comparative Analyses of Reference Genomes

The downloaded genome isolates from NCBI included the reference genomes. These reference genome strains were hilgardii_FLUB and oeni_AWRIB429. Comparative analyses using Rapid Annotations using Subsystems Technology (RAST) (Aziz et al. 2008) server were performed in order to detect which pathway was different between the two strains. RAST tool kit (RASTtk) was used as the annotation scheme (Brettin et al. 2015). The outputs of these analyses were compared manually to identify differences in terms of pathway classes between the two strains. The number of genes associated with specific pathway classes was normalised in relative abundance to facilitate result interpretation. Two distinct graphs were generated using Microsoft Excel's hierarchy chart graphical option.

### 2.3 Potential Reaction of the Two Core‐Genome Species

The exploration of potential reactions was conducted through the bioinformatic platform Kbase software (Arkin et al. 2018), utilising the two core genomes as input data. This software offers a suite of tools for genomic data analysis, including genome annotation, metabolic modelling and comparative genomics. In our project, we applied genome‐scale metabolic models using the Model SEED (Henry et al. 2010) tool, specifically employing the ‘Build Multiple Metabolic Models’ function (Henry et al. 2010) in two distinct modes. The Model SEED tool was tasked with elucidating the potential reactions that each genome could undertake, taking into account the environmental conditions in which the bacteria express their functions. Consequently, two distinct media were employed for the analyses.

Firstly, the default medium was utilised, without nutrient limitations, to observe the comprehensive range of potential reactions specific to each species. This medium was the default one in the Model SEED tool named Complete media. The Complete media represents an abstraction of the compounds available in the biochemistry database. It includes every compound that can be transported into the extracellular compartment, or in simpler terms, any compound for which a transport reaction is available, is included in the Complete media. This list is dynamically generated in real time, meaning that whenever flux balance analysis is run with Complete media, the available transporters are extracted from the media database. This allowed us to assess the full metabolic capabilities of the bacteria under optimal growth conditions. The Complete media is also used for the analyses of co‐inoculation between *L. hilgardii* or *O. oeni* together with the *Saccharomyces cerevisiae* EC1118 to observe the differential reaction that occurs under anaerobic conditions between the two couples in order to simulate the co‐inoculation in the vinification context. Secondly, a synthetic grape juice medium (SGJM) was employed to simulate a post‐alcoholic fermentation scenario, replicating the conditions of grape must as outlined in (Wang et al. 2003), This medium was chosen to mimic the environmental conditions encountered by the bacteria during the fermentation process in a winemaking context.

By utilising these two distinct media, we aimed to gain insight into the metabolic responses of the bacteria under different environmental conditions, shedding light on their potential metabolic pathways and reactions in both ideal and realistic settings. Reactions that are described in Tables 1 and 2.

**TABLE 1:** The table shows the main enzymatic findings related to winemaking looking at the pangenome of the two LAB.

Found in	Enzyme	Function	Role in winemaking
Common	Glycosylases	Hydrolyzing carbohydrates	Volatile aromatic molecules production and colour stabilisation
Common	Diacetyl reductase	Converting diacetyl into acetoin	Buttery aroma production
*L. hilgardii*	Thiol peroxidase	Breakdown of volatile thiophenols	Smoke aroma production
*L. hilgardii*	Cysteine proteases	Peptide bond cleavage in proteins	Wine stability improvement
Common	Carbamoyl‐phosphate synthase	Synthesis of carbamyl phosphate	Ethyl carbamate production
Common	Arginine deaminase	Break down arginine	Ethyl carbamate production
*L. hilgardii*	Ethanolamine ammonia‐lyase	Synthesis of ethanolamine	Ethanolamine production
*L. hilgardii*	Agmatine deiminase	Synthesis of agmatine	Putrescine production

*Note:* ‘Common’ means that the enzyme is found in both bacteria. ‘ *L. hilgardii* ’ means that the enzyme is unique to this bacteria.

**TABLE 2:** The table presents potential reactions initiated by *L. hilgardii* and not by *O. oeni* within an *in silico* synthetic Complete medium.

Reaction id	Enzyme	Reaction	EC	Potential role in winemaking
R00192	S‐Adenosyl‐L‐homocysteine hydrolase	H_2_O + S‐Adenosyl‐homocysteine → Homocysteine + Adenosine	EC 3.13.2.1_;L1_Hydrolases;L2_Acting on carbon‐sulfur bonds;L3_Thioether and trialkylsulfonium hydrolases	Improve the mouthfeel
R00253	L‐Glutamate:ammonia ligase (ADP‐forming)	ATP + NH_3_ + L‐Glutamate → ADP + Phosphate + L‐Glutamine + H^+^	EC 6.3.1.2_;L1_Ligases;L2_Forming carbon‐nitrogen bonds;L3_Acid—ammonia (or amine) ligases (amide synthases);L4_glutamine synthetase	Unkonw
R00342	(S)‐malate:NAD^+^ oxidoreductase	NAD + L‐Malate → NADH + Oxaloacetate + H^+^	EC 1.1.1.37_;L1_Oxidoreductases;L2_Acting on the CH‐OH group of donors;L3_With NAD+ or NADP+ as acceptor;L4_malate dehydrogenase	Unkonw
R00346	Diphosphate:oxaloacetate carboxy‐lyase (transphosphorylating;phosphoenolpyruvate‐forming)	PPi + Oxaloacetate → Phosphate + CO_2_ + Phosphoenolpyruvate	EC 4.1.1.38_;L1_Lyases;L2_Carbon‐carbon lyases;L3_Carboxy‐lyases;L4_phosphoenolpyruvate carboxykinase (diphosphate)	Unkonw
R00351	Acetyl‐CoA:oxaloacetate C‐acetyltransferase (thioester‐hydrolysing)	CoA + H^+^ + Citrate ← H_2_O + Acetyl‐CoA + Oxaloacetate	EC 2.3.3.1_;L1_Transferases;L2_Acyltransferases;L3_Acyl groups converted into alkyl groups on transfer;L4_citrate (Si)‐synthase	Unkonw
R00405	Succinate:CoA ligase (ADP‐forming)	ATP + CoA + Succinate ← ADP + Phosphate + Succinyl‐CoA	EC 6.2.1.5_;L1_Ligases;L2_Forming carbon‐sulfur bonds;L3_Acid‐thiol ligases;L4_succinate—CoA ligase (ADP‐forming)	Increases the sour salty and bitter teste (Torres‐Guardado et al. 2022)
R00709	Isocitrate:NAD^+^ oxidoreductase (decarboxylating)	NAD + Isocitrate → NADH + CO_2_ + 2‐Oxoglutarate	EC 1.1.1.41_;L1_Oxidoreductases;L2_Acting on the CH‐OH group of donors;L3_With NAD+ or NADP+ as acceptor	Influence the structure and balance
R00830	Succinyl‐CoA:glycine C‐succinyltransferase (decarboxylating)	Glycine + H+ + Succinyl‐CoA → CoA + CO_2_ + 5‐Aminolevulinate	EC 2.3.1.37_;L1_Transferases;L2_Acyltransferases;L3_Transferring groups other than aminoacyl groups;L4_5‐aminolevulinate synthase	Unkonw
R00959	Alpha‐D‐Glucose 1‐phosphate 1,6‐phosphomutase	Glucose‐1‐phosphate ← D‐glucose‐6‐phosphate	EC 5.4.2.2_;L1_Isomerases;L2_Intramolecular transferases;L3_Phosphotransferases (phosphomutases)	Carbohydrate metabolism
R01082	(S)‐malate hydro‐lyase (fumarate‐forming)	L‐Malate ← H_2_O + Fumarate	EC 4.2.1.2_;L1_Lyases;L2_Carbon‐oxygen lyases;L3_Hydro‐lyases;L4_fumarate hydratase	increases the sour salty and bitter teste (Torres‐Guardado et al. 2022)
R01150	D‐alanine:D‐alanine ligase (ADP‐forming)	ATP + (2) D‐Alanine → ADP + Phosphate + H^+^ + Ala‐Ala	EC 6.3.2.4_;L1_Ligases;L2_Forming carbon‐nitrogen bonds;L3_Acid—amino‐acid ligases (peptide synthases);L4_D‐alanine—D‐alanine ligase	Enhance the resilience of LAB in harsh environment
R01324	Citrate hydroxymutase	Citrate → Isocitrate	EC 4.2.1.3_;L1_Lyases;L2_Carbon‐oxygen lyases;L3_Hydro‐lyases;L4_aconitate hydratase	Increase aroma complexity
R01513	3‐Phospho‐D‐glycerate:NAD^+^ 2‐oxidoreductase	NAD + 3‐Phosphoglycerate ↔ NADH + H^+^ + 3‐Phosphonooxypyruvate	EC 1.1.1.95_;L1_Oxidoreductases;L2_Acting on the CH‐OH group of donors;L3_With NAD+ or NADP+ as acceptor;L4_phosphoglycerate dehydrogenase	Carbohydrate metabolism
R08549	2‐Oxoglutarate dehydrogenase complex	NAD + CoA + 2‐Oxoglutarate → NADH + CO_2_ + Succinyl‐CoA	EC 1.2.1.105_;L1_Oxidoreductases;L2_Acting on the aldehyde or oxo group of donors;L3_With NAD+ or NADP+ as acceptor;L4_2‐oxoglutarate dehydrogenase system	Antioxidative defence

*Note:* It includes key information such as the ‘Reaction ID’, the catalysing enzyme, the complete reaction representation, and the Enzyme Commission (EC) number, providing a comprehensive classification of the enzyme involved (level 1, level 2 and level 3). The numerical values within the brackets denote the stoichiometry of the reaction.

### 2.4 Malolactic Enzyme Molecular Docking

The SWISS‐MODEL (Waterhouse et al. 2018), a fully automated protein structure homology‐modelling server, was used to model the 3‐dimensional configuration of the protein starting from the aminoacidic sequencing extracted from the pangenome of the two species: *L. hilgardii* and *O. oeni* . Following the finding of the molecular templates, the affinity with the ligand malic acid by the Vina score has been calculated in order to understand which different structure could be more affine to the ligand.

This task was performed using CB‐Dock (Liu et al. 2020) a protein‐ligand docking method that automatically identifies the binding sites, calculates the center and size, customises the docking box size according to the query ligands and then performs the molecular docking with AutoDock Vina.

### 2.5 Malolactic Convertion in Synthetic Wine‐Like Medium

All reagents and materials used in this study were of analytical grade. L‐malic acid, D‐fructose, glucose and ethanol were purchased from Sigma‐Aldrich (St. Louis, MO, USA). Yeast extract, peptone and MRS broth components were obtained from Oxoid (Basingstoke, UK). Synthetic wine‐like medium (SWM) was prepared using tartaric acid (Merck, Darmstadt, Germany), potassium bitartrate (Sigma‐Aldrich) and ethanol (Carlo Erba, Milan, Italy). The strains *Lentilactobacillus hilgardii* DSM 20176ᵀ and *Oenococcus oeni* DSM 20252ᵀ were obtained from the DSMZ culture collection (Braunschweig, Germany). All culture media were sterilised by autoclaving (121°C for 15 min) and stored at 4°C until use. Water used in all experiments was ultrapure grade (Milli‐Q system, Millipore, Burlington, MA, USA).

*Lentilactobacillus hilgardii* DSM 20176^T^ was cultured for 3 days at 30°C in de Man, Rogosa and Sharpe (MRS; Difco) broth. Following initial growth, the culture was inoculated at 1% (v/v) into MRS supplemented with 10% (v/v) ethanol to allow for acclimatisation to ethanol stress.

*Oenococcus oeni* DSM 20252^T^ was grown in MRS broth supplemented with 10 g/L L‐malic acid, pH 4.8. After 7 days of incubation at 30°C, the culture was transferred at 1% (v/v) into the same modified MRS medium, now supplemented with 10% (v/v) ethanol.

After 4 days of incubation under these conditions, both cultures were standardised by flow cytometry. Samples were stained with 0.1 μM SYTO 24 (Thermo Fisher Scientific) and 0.2 μM propidium iodide (PI; Sigma‐Aldrich), then incubated in the dark at 37°C for 15 min. Flow cytometric analysis was performed using a BD Accuri C6 Plus flow cytometer (BD Biosciences, Milan, Italy) with thresholds set at FSC‐H 3000 and SSC‐H 1000. All parameters were recorded as logarithmic signals. Green fluorescence (SYTO 24) and red fluorescence (PI) were detected in FL1 (excitation 488 nm, emission 530/30 nm) and FL3 (excitation 488 nm, emission 670 LP nm) channels, respectively. Electronic gating on the SYTO 24/PI density plot was used to determine total bacterial concentration (events/mL), active fluorescent units (AFU) and non‐active fluorescent units (nAFU), in accordance with ISO 19344:2015.

The standardised bacterial suspensions (1 × 10^7 AFU/mL) were inoculated into 10 mL of a synthetic wine‐like medium (SWM). This medium was adapted from Olguín et al. (2009) with modifications and consisted of (per litre): 2.5 g casamino acids, 2.5 g peptone, 2 g fructose, 0.5 g trehalose, 0.5 g sodium citrate, 2 g L‐malic acid, 2 g L‐tartaric acid, 5 mL glycerol, 0.28 g sodium acetate, 0.6 g KH_2_PO_4_, 0.13 g MgSO_4_·7H_2_O, 0.03 g MnSO_4_·H_2_O, 0.13 g CaCl_2_ and 0.45 g KCl. The pH was adjusted to 3.4, and the ethanol content was 12% (v/v). Inoculated media were incubated under anaerobic conditions at both 25°C and 10°C.

The L‐malic acid as well as the L‐lactic and the fructose have been quantified by an enzymatic kit (R‐Biopharm, Darmstadt, Germany).

### 2.6 Statistical Analyses

All experimental data are presented as mean ± standard deviation (SD) from three independent biological replicates, each performed in technical triplicate. Statistical analyses were performed using R version 4.3.1. Differences between groups were assessed by one‐way ANOVA followed by Tukey's post hoc test for multiple comparisons. When only two groups were compared, an unpaired two‐tailed Student's *t*‐test was applied. A *p*‐value < 0.05 was considered statistically significant. Different lowercase letters in the figures indicate statistically significant differences among means according to Tukey's test.

## 3 Results and Discussion

### 3.1 Pangenome of Lentilactobacillus Hilgardii and *Oenococcus oeni*

**FIGURE 1:**
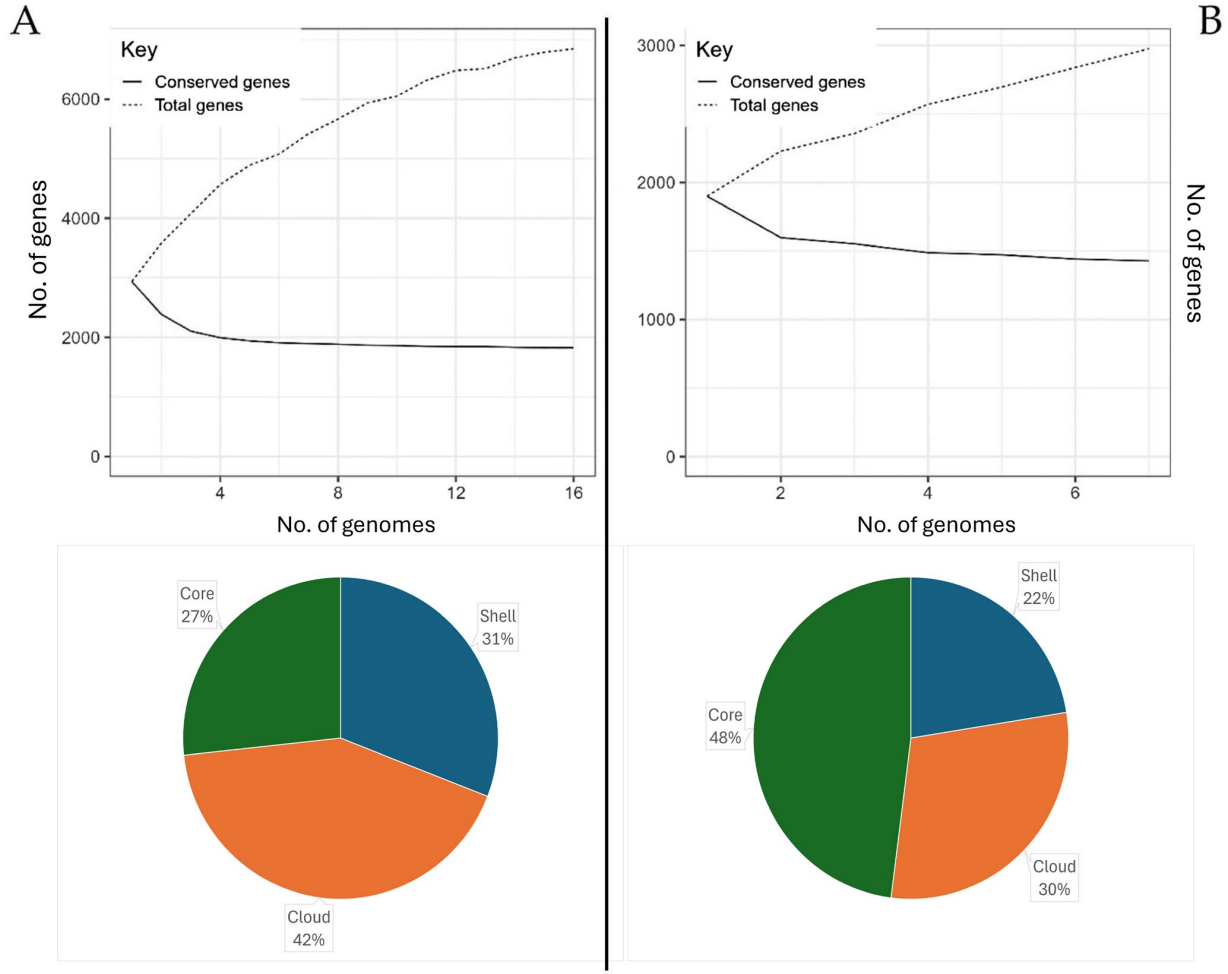
Pangenome results for *L. hilgardii* and *O. oeni* . (A) Calculation of core‐ and pan‐genome sizes using 16 strains of the *L. hilgardii* species. Graph showing the proportion of conserved genes relative to the total number of genes across a set of 16 genomes. Pie chart with the division of the genes present in the pangenome based on the level of sharing of the specific genes between the genomes of *L. hilgardii* . (B) Calculation of core‐ and pan‐genome sizes using 7 strains of the *O. oeni* species. Graph showing the proportion of conserved genes relative to the total number of genes across a set of 7 genomes. Pie chart with the division of the genes present in the pangenome based on the level of sharing of the specific genes between the genomes of *O. oeni* .

In this study, all available genomes from NCBI of *L. hilgardii* (*n* = 16) were considered, and the pangenome was analysed to identify the core genes of this species. In particular, it has been found 1828 genes to be shared among all 16 genomes, forming the core genome of *L. hilgardii* (Figure 1a). Notably, it has been found 2904 genes to be unique and distributed across various genomes of *L. hilgardii* , indicating a high level of genetic heterogeneity of this species. Pangenome analysis was also conducted for the species *Oenococcus oeni* , using only complete genomes as a reference (*n* = 7) available on NCBI. In *O. oeni* , 1428 core genes were identified, while 880 single genes were present in various strains (Figure 1b).

Using this data, a core‐genome was constructed, composed only of genes shared from all the strains belonging to a species for both species, to observe the differences between them. Enzymes related to MLF were identified as shared between the two genera, including the crucial malolactic enzyme.

### 3.2 Differences in Malolactic Enzyme Between the Two Species

**FIGURE 2:**
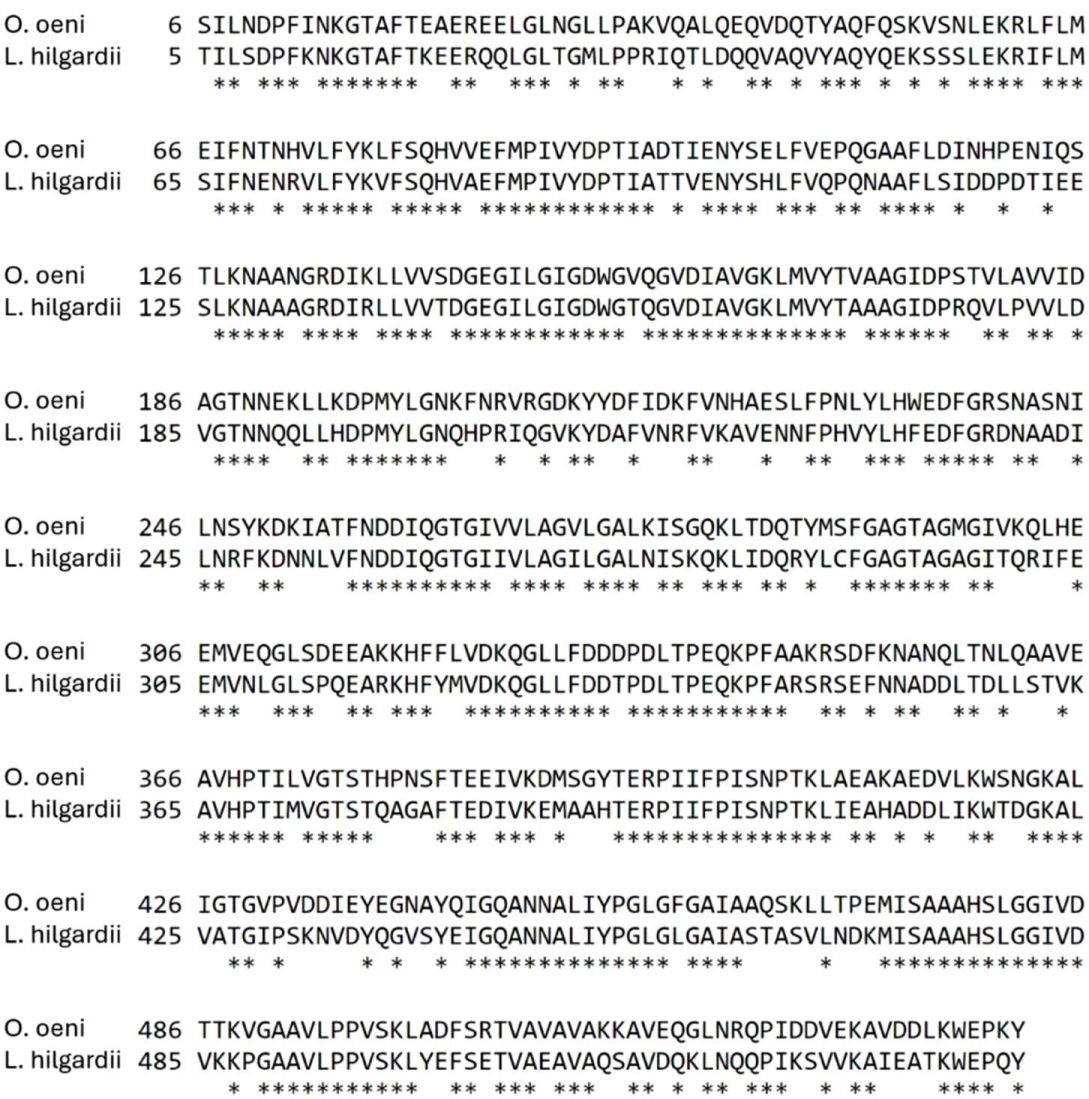
Alignment of the malolactic enzyme between the two species *O. oeni* (first row) and *L. hilgardii* (second row). The percent of identity is 67.9% in 536 residues overlap; Score: 1935.0; Gap frequency: 0.0%. The stars highlight the same residue between the two sequences.

The primary enzyme anticipated from bacteria engaged in secondary fermentation is the malolactic enzyme. A comparison of the amino acid sequences of the malolactic enzyme from *O. oeni* and *L. hilgardii* revealed a significant degree of identity, with 67.9% similarity observed, as depicted in Figure 2.

**FIGURE 3:**
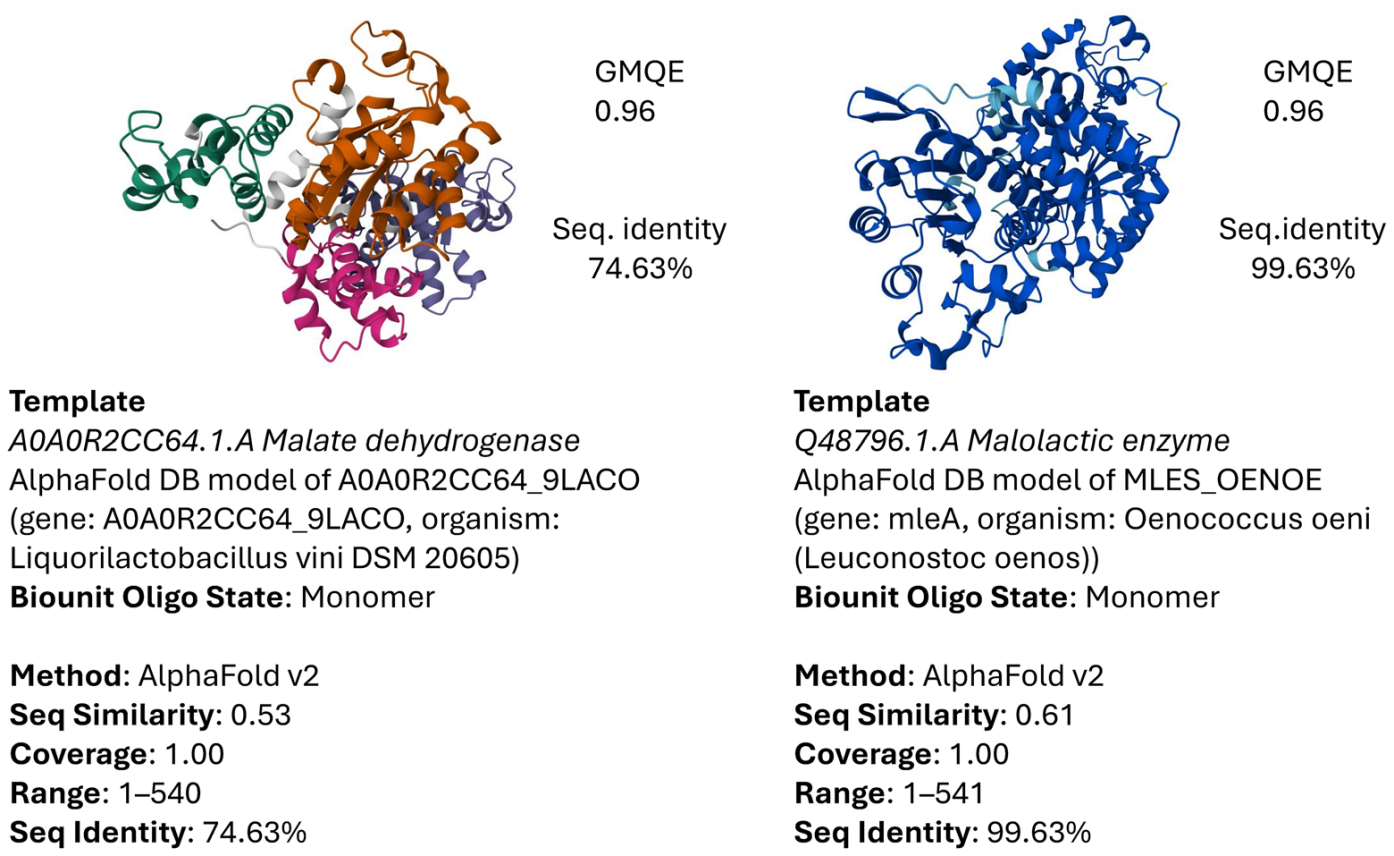
Templates obtained by SWISS‐MODEL. Template A0A0R2CC64.1.A for *L. hilgardii* ; template Q48796.1.A for *O. oeni* .

Based on the protein sequences, the 3‐dimensional structure of a protein sharing similarities with two enzymes from the UniProt database was modelled: one corresponding to *L. hilgardii* , with a sequence identity of 74.63%, and another to *O. oeni* , with a sequence identity of 99.63% (Figure 3). Utilising these templates, CB Docking was employed to assess the affinity for malic acid. Surprisingly, the obtained results in terms of ‘Vina score’ were identical, both yielding a score of −4.9. This indicates that despite differences in amino acid sequences and 3D structures, the ligand's affinity with the enzyme remains constant. The consistent Vina scores obtained for the affinity of malic acid with the two enzymes, despite differences in their amino acid sequences and 3D structures, raise intriguing questions about ligand–enzyme interactions. This phenomenon suggests that certain key binding residues or structural motifs, shared between the enzymes, may be responsible for the observed affinity.

### 3.3 Comparative Analysis of Fermentative Performance of *Oenococcus oeni* and Lentilactobacillus Hilgardii at Different Temperatures

**FIGURE 4:**
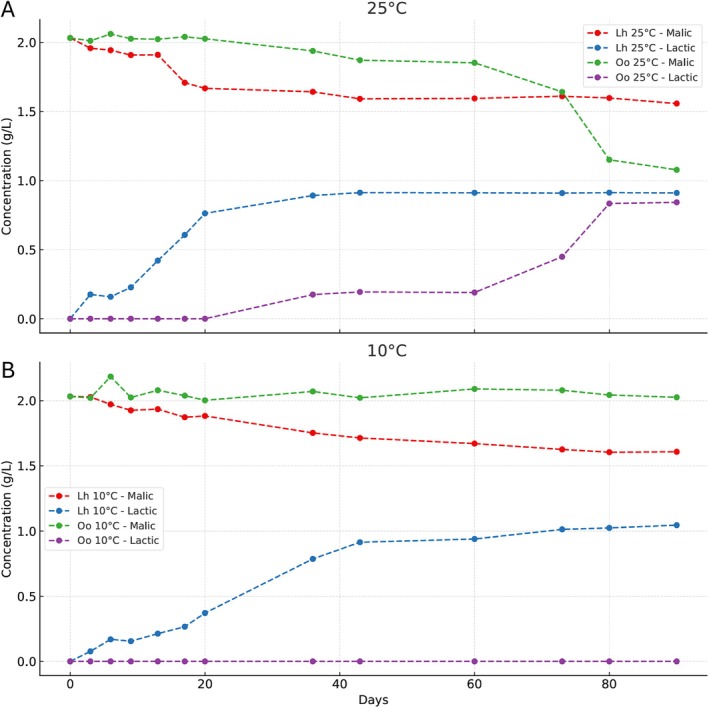
Reduction of L‐malic acid and production of L‐lactic acid by *Oenococcus oeni* and *Lentilactobacillus hilgardii* at two incubation temperatures. The graphs display the concentrations of L‐malic acid (g/L) and L‐lactic acid (g/L) over time (days) in wine samples inoculated with *O. oeni* or *L. hilgardii* , and incubated at 25°C (A) or 10°C (B). Data represent the mean of three biological replicates; error bars indicate standard deviation. At 25°C, no statistically significant difference was observed between the strains (*p* = n.s.), while at 10°C, *L. hilgardii* showed significantly faster L‐malic acid reduction and L‐lactic acid production compared to *O. oeni* (*p* < 0.001). Different letters indicate statistically significant differences among means according to Tukey's post hoc test (*p* < 0.05).

The progression of MLF by *Oenococcus oeni* DSM20252 and *Lentilactobacillus hilgardii* DSM20176 was monitored by quantifying L‐malic acid reduction and L‐lactic production at two temperatures, 25°C and 10°C, over a period of 90 days. The observed trends (Figure 4A) demonstrate significant differences in the metabolic performance of the two species under these conditions, particularly in relation to temperature sensitivity and efficiency in malic acid reduction.

At 25°C, both strains were capable of degrading L‐malic acid with L‐lactic production, although with different kinetics and efficiency. *O. oeni* showed a relatively slow but progressive reduction, with L‐malic acid levels decreasing from 2.033 g/L ± 0.047 at Day 0 to 1.078 g/L ± 0.238 by Day 90 and the L‐lactic increasing from 0 g/L at Day 0 to 0.843 g/L ± 0.313 by Day 90. During the first 20 days, fluctuations in malic acid concentration (e.g., 2.062 g/L ± 0.026 on Day 6 and 2.041 g/L ± 0.001 on Day 17) suggest a delayed onset of malolactic activity, possibly due to metabolic adaptation or analytical variability. Malolactic conversion began after Day 20, consistent with the known adaptation period of *O. oeni* in wine‐like conditions (Versari et al. 1999; Bartowsky and Henschke 2004; Liu 2002; du Toit et al. 2011). Despite this, neither strain completed the complete reduction of malic acid by Day 90, likely due to nutritional limitations or inhibition of the end product.

At 10°C (Figure 4B), the malolactic conversion by *O. oeni* was markedly impaired. L‐malic acid levels remained essentially unchanged throughout the trial, with only minor fluctuations ranging from 2.033 ± 0.047 to 2.090 ± 0.018 g/L. These variations fall within the range of analytical variability and do not indicate any biologically meaningful reduction, consistent with the absence of detectable L‐lactic acid production. This behaviour reflects the psychrotolerant but slow‐growing nature of *O. oeni* , whose metabolic activity is significantly reduced at low temperatures (Lonvaud‐Funel 1999; Costello et al. 2003). In contrast, *L. hilgardii* retained partial activity at 10°C, with L‐malic acid levels decreasing from 2.033 g/L ± 0.047 to 1.608 g/L ± 0.006 by Day 90 with L‐lactic increasing from 0 to 1.045 g/L ± 0.013 by Day 90. This suggests that *L. hilgardii* possesses a broader temperature tolerance and retains sufficient enzymatic activity for partial MLF even at low temperatures. Previous studies have reported strain‐specific cryotolerance among lactobacilli, particularly those isolated from environments with fluctuating temperatures (Bâati et al. 2000; Gautier et al. 2013).

**FIGURE 5:**
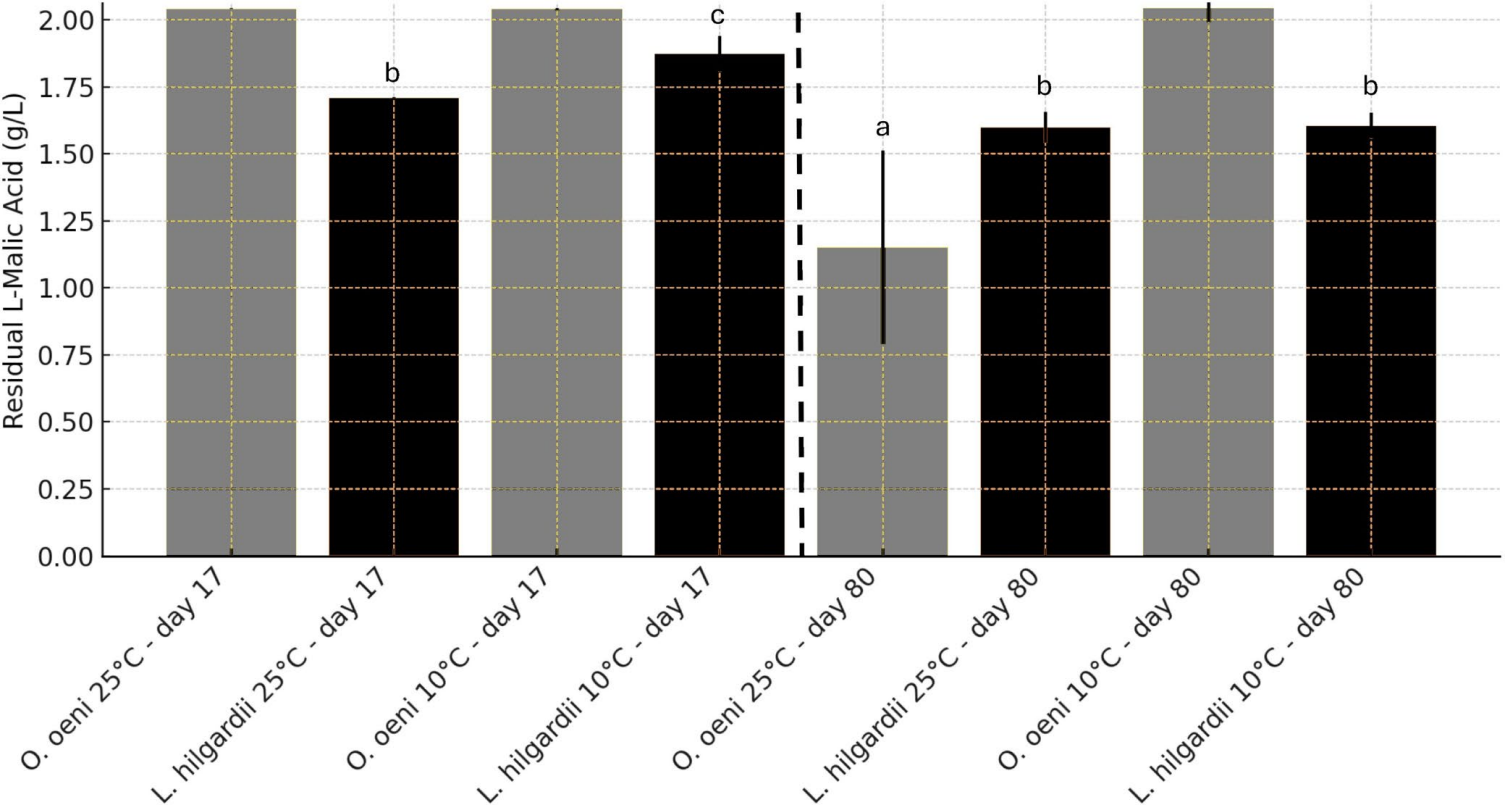
Fructose concentration (g/L) at Day 0 and Day 90 during fermentation with *Lentilactobacillus hilgardii* and *Oenococcus oeni* at 25°C and 10°C. Data are presented as mean ± standard deviation (*n* = 3). *L. hilgardii* exhibited a markedly higher capacity for fructose consumption under both thermal conditions, whereas *O. oeni* showed limited or no utilisation, particularly at low temperature.

Moreover, fructose levels were measured at both the beginning and the end of the fermentation period to assess non‐malofermentative metabolic activity (Figure 5). Notably, *L. hilgardii* was able to consume nearly all the available fructose, with concentrations dropping from 2.430 g/L ± 0.042 at Day 0 to 0.040 g/L ± 0.003 and 0.052 g/L ± 0.003 at 25°C and 10°C, respectively, by Day 90. In contrast, *O. oeni* exhibited limited or no fructose consumption, with final concentrations of 1.984 g/L ± 0.045 at 25°C and 2.430 g/L ± 0.043 at 10°C. These results support the notion that *L. hilgardii* , beyond its malolactic potential, maintains a higher fermentative activity under both thermal conditions, likely due to a more versatile carbohydrate metabolism.

While *O. oeni* remains the conventional choice for malolactic fermentation in winemaking, the observed limitations at low temperature suggest that its application may be constrained in cooler fermentation settings or in styles where early MLF is desirable. On the other hand, *L. hilgardii* demonstrates promising potential for low‐temperature fermentations, combining partial malolactic conversion with effective fructose utilisation. Further strain selection and phenotypic characterisation could enhance the exploitation of *L. hilgardii* as a candidate for controlled MLF under non‐conventional enological conditions.

### 3.4 Enzymatic Potential Concerning the Overall Quality of Wine

Both strains commonly possess glycosylases as well, a family of enzymes responsible for hydrolyzing carbohydrates from a various diversity of molecules. Glycosylases are also known to be key enzymes in forming volatile aromatic molecules or stabilising the colour of wine (Devi et al. 2020; Caffrey and Ebeler 2021). This linkage underscores the broader implications of the identified enzymes in shaping both MLF processes and the overall quality attributes of wine. Regarding aromatic molecules, the enzyme diacetyl reductase, responsible for buttery aroma formation from diacetyl and acetoin molecules, was also found in common (Bartowsky and Henschke 2004). Specifically, diacetyl reductase is essential for converting diacetyl into acetoin, through the conversion of NADH(P) in NAD(P). Diacetyl arises from the chemical oxidative decarboxylation of α‐acetolactate. The next step is the reduction of acetoin in 2,3‐butanediol helping to maintain the redox balance in cells with the oxidation of NADH in NAD. In LAB, the metabolism of 1 mol of citrate typically yields 1 mol of acetic acid, 2 mol of carbon dioxide, and 0.5 mol of diacetyl, acetoin and 2,3‐butanediol combined. This process significantly influences the wine's aroma profile while contributing to cellular redox equilibrium (Bartowsky and Henschke 2004).

Concerning the enzymes present only in *L. hilgardii* the thiol peroxidase has been found, known to act as an antioxidant by removing peroxides or H_2_O_2_ in the periplasmic space of catalase and peroxidase‐deficient *Escherichia coli* (Cha et al. 1995). The presence of thiol peroxidase, specifically linked to the breakdown of volatile thiophenols, highlights the potential contribution of these enzymes to the overall aromatic profile, with implications for the discernment and characterisation of smoke aromas in wine (Tomasino et al. 2023). Additionally, cysteine proteases, exclusive to *L. hilgardii* , contain the cysteine residue in their active site and are involved in peptide bond cleavage in proteins, playing a significant role in the winemaking process and wine aging. During wine aging, proteins can precipitate, forming deposits known as ‘protein sediment’. Enzymes like cysteine proteases can contribute to reducing these deposits, improving wine stability suggesting their employmentas clarification agents to eliminate suspended particles and enhance wine clarity (Benucci et al. 2016).

In summary, the shared and unique enzymatic activities of LAB strains, such as glycosylases and diacetyl reductase, play crucial roles in shaping the aroma, colour stability and overall quality of wine. Additionally, enzymes exclusive to *L. hilgardii* , such as thiol peroxidase and cysteine proteases, might contribute to antioxidant functions and protein stability, further influencing wine clarity and aging.

### 3.5 Enzymatic Potential Concerning the Safety of Wine

The carbamoyl‐phosphate synthase and the arginine deaminase were both enzymes found in common between the two species. Urea is a byproduct of arginine metabolism by yeast, and it is the primary precursor in the production of ethyl carbamate in wine. However, ethyl carbamate levels may also rise following MLF (Masqué et al. 2011). Certain strains of LAB possess the ability to break down arginine found in grape must and wine through the arginine deiminase pathway. This enzymatic process yields citrulline and carbamyl phosphate. In acidic conditions, both compounds can react with ethanol, resulting in the formation of ethyl carbamate, whose carcinogenic properties are well‐documented (Masqué et al. 2011).

From the enzymatic potential examination of *L. hilgardii* , based on the core genome, the presence of enzymes involved in the production of biogenic amines, specifically ethanolamine, putrescine and histamine has been revealed. Accordingly, *Lentilactobacillus hilgardii* X_1_B, isolated from wine was able to produce putrescine via the agmatine deaminase pathway (Alberto et al. 2007). Interestingly, the levels of putrescine were reduced when phenolic compounds were added to synthetic basal medium, suggesting that their occurrence in wine could partially control the formation of some biogenic amines (Alberto et al. 2007). Furthermore, putrescine is also known to enhance the toxicity of histamine mainly by competitively inhibiting the diamine oxidases involved in biogenic amines detoxification at gut level (Sánchez‐Pérez et al. 2022). In this context, enzymes for the synthesis of spermidine and putrescine, although found in *L. hilgardii* , were also present in the core genome of *O. oeni* . The Histidinol Dehydrogenase (HDH) is an enzyme crucial for the biosynthesis of histidine. This enzyme, found exclusively in the core genome of *L. hilgardii* , catalyses the four‐electron oxidation of L‐histidinol to histidine. This process involves sequential NAD‐dependent oxidations: first, L‐histidinol is converted to L‐histidinaldehyde, and then to L‐histidine. After decarboxylation, L‐histidine is converted into the biogenic amine histamine (Köhler et al. 2016) even though the decarboxylase was not found in the core genome of this bacterium. In the literature, there are references to *L. hilgardii* 's potential to produce tyramine (Moreno‐Arribas et al. 2000). However, our analysis of the core genome did not confirm this as a universally present trait, suggesting it may be strain‐specific.

In summary, careful management of microbial activities is crucial for maintaining wine safety. Enzymes responsible for the production of biogenic amines are either shared with or unique to *L. hilgardii* (Table 1), necessitating particular attention to this species. It is important to emphasise that biogenic amine production is strain‐specific. Therefore, sequencing more strains is essential to determine whether biogenic amine production is a characteristic of the *entire L. hilgardii* species.

### 3.6 Enzymatic Potential Concerning the Stress Tollerance

In the core genomes of both organisms, elements inherent to the stress response have been identified. While several putative universal stress proteins are commonly found in both, a distinctive feature of *L. hilgardii* is the presence of the peroxide stress resistance protein YaaA. This protein plays a crucial role in the stress response to hydrogen peroxide (H_2_O_2_). YaaA mitigates the harmful effects of peroxide stress by reducing intracellular iron levels, thereby attenuating the Fenton reaction, which can lead to significant DNA damage (Liu et al. 2011). Despite its importance, the precise molecular mechanism through which YaaA operates remains unknown. Unique in *L. hilgardii* is also the Heat shock protein 15 and the Heat‐inducible transcription repressor HrcA. HrcA plays a crucial role in regulating gene expression during temperature stress. It controls the transcription of *groE* and *dnaK* operons by binding to the palindromic CIRCE element, likely as a dimer. Interestingly, the activity of the HrcA repressor is modulated by GroE chaperones (Liu et al. 2005; Spano and Massa 2006; Darsonval et al. 2018). Moreover the Alkaline shock protein 23 is present only in *L. hilgardii* . It is a member of the Pfam DUF322 family of proteins and was first identified in *S. aureus* as a 23 kDa protein that is significantly enhanced upon a pH upshift from 7 to 10 (Kuroda et al. 1995).

Unlike *L. hilgardii* , CtsR has been identified in the genome of *O. oeni* as the only regulator of genes typically related to stress response (Grandvalet et al. 2005). The small heat shock protein Lo18 has been reported to be involved in the adaptation of *O. oeni* to ethanol stress by preventing thermal aggregation of proteins and playing a crucial role in membrane quality control (Maitre et al. 2014). One unique feature of *Oenococcus oeni* is the presence of the Cold Shock‐like Protein CspLA. This small protein is particularly significant in bacteria for its role in response to rapid temperature downshifts, commonly known as cold shock. When a cell undergoes cold shock, several physiological changes occur: membrane fluidity decreases, enzyme activity is compromised, and the efficiency of transcription and translation is reduced due to the stabilisation of nucleic acid secondary structures. CspLA is transiently activated during these temperature shifts, aiding the cell in adapting to sudden changes in environmental conditions (Roy and Ray 2023).

### 3.7 Metabolic Prediction in Synthetic Rich Medium or Post‐Alcoholic Fermentation (AF) Medium

To extrapolate the metabolism prediction of *L. hilgardii* and *O. oeni* genomes formed only by the core genes of their respective species, a genome‐scale metabolic model is applied using *in silico* rich medium without nutritional limitations keeping the anaerobic condition referred to in this manuscript as Complete media. This approach aimed to avoid restrictions on collecting data related to the metabolic potential. Following this, we utilised a virtual medium, the synthetic grape juice medium (SGJM), designed to mimic wine, in order to simulate the conditions resembling the wine at the conclusion of alcoholic fermentation (Wang et al. 2003). Differences between the two species were then analysed.

When using the Complete media, several differences in terms of potential reactions were observed in *L. hilgardii* compared with *O. oeni* . Among these, the most significant differences were related to an isomerase, three lyases and two oxidoreductases. Specifically, the enzymes mentioned are represented in Table 2.

The alpha‐D‐glucose 1‐phosphate 1,6‐phosphomutase and the 3‐phospho‐D‐glycerate:NAD+ 2‐oxidoreductase found in *L. hilgardii* in the context of winemaking can be crucial, as they play key roles in carbohydrate metabolism. For instance, the (S)‐malate hydro‐lyase (fumarate‐forming) enzyme is involved in the citric acid cycle under anaerobic conditions: the reverse reductive citric acid cycle, ultimately aiming to produce succinate (Camarasa et al. 2003). In relation to citrate metabolism, have been identified citrate hydroxymutase and isocitrate:NAD+ oxidoreductase enzymes, unique to *L. hilgardii* . These enzymes specifically act on citrate, a substrate utilised by LAB during MLF, and isocitrate, respectively. Citrate metabolism could enhance wine aroma and increase aroma complexity by producing various volatile compounds, such as acetate, diacetyl, acetoin and butanediol (Cappello et al. 2017). Isocitrate: NAD+ oxidoreductase converts isocitrate to alpha‐ketoglutarate, indirectly influencing the production of organic acids like citric acid. Variations in the concentration of organic acids, including those produced through the Krebs cycle, can influence the structure and balance of the wine, contributing to its aromatic and organoleptic complexity (Chidi et al. 2018). Another enzyme involved in the Krebs cycle found in *L. hilgardii* 's metabolic potential is the 2‐oxoglutarate dehydrogenase complex, which utilises 2‐oxoglutarate, also known as α‐ketoglutaric acid or 2‐oxoglutaric acid, a key intermediate in the Krebs cycle. It is produced by the deamination of glutamate. This compound participates in various biological processes, including antioxidative defence (Long and Halliwell 2011). The 2‐oxoglutarate is a secondary product of fermentation and plays an essential role in binding with sulfur compounds (Herzan et al. 2020). The volume of 2‐oxoglutaric acid is almost 40 mg/L in unsulfured must compared to sulfured samples before fermentation. Therefore, the presence of SO_2_ during fermentation causes the microorganisms to produce less 2‐oxoglutarate. A study has found that during wine aging, the value of 2‐oxoglutarate is relatively stable when the must is treated with SO_2_. Conversely, in the absence of SO_2_ during fermentation, the concentration of 2‐oxoglutarate is higher, and its content varies considerably during wine aging (Herzan et al. 2020). Of particular interest is an enzyme associated with bacterial growth in challenging conditions, characteristic of environments like wine. The D‐alanine ligase (ADP‐forming), has the potential to enhance the resilience of LAB in harsh environments, such as wine, by playing a crucial role in bacterial cell wall formation (Pederick et al. 2020).

Regarding the prediction of reactions occurring in glucose‐depleted medium with a high concentration of ethanol (13% ABV) due to alcoholic fermentation, fewer results were obtained, as expected. Many of the reactions were the same as those predicted with the Complete media without nutritional limitations. Six oxidoreductases, two transferases, two lyases and one ligase were found (Table 3). Different from the prediction in a complete nutrient medium, the reactions guided by (S)‐lactate:ferricytochrome‐c 2‐oxidoreductase and (S)‐Lactate:NAD+ oxidoreductase were found. Both are involved in the MLF after alcoholic fermentation, corresponding to reactions R00196 and R00703, respectively in Table 3. These reactions shed light on potentially novel pathways within MLF, warranting further exploration and understanding in the context of wine production.

**TABLE 3:** The table presents potential reactions initiated by *L. hilgardii* and not in *O. oeni* within an in silico synthetic medium post‐alcoholic fermentation.

Reaction id	Enzyme	Reaction	EC	Potential role in winemaking
R00196	(S)‐Lactate:ferricytochrome‐c 2‐oxidoreductase	(2) Cytochrome c3^+^ + L‐Lactate ↔ Pyruvate + (2) H^+^ + (2) Cytochrome c2^+^	EC 1.1.2.3_;L1_Oxidoreductase reactions;L2_Acting on the CH‐OH group of donors;L3_With a cytochrome as acceptor	Unknown
R00342	(S)‐malate:NAD+ oxidoreductase	NAD + L‐Malate → NADH + Oxaloacetate + H^+^	EC 1.1.1.37_;L1_Oxidoreductase reactions;L2_Acting on the CH‐OH group of donors;L3_With NAD+ or NADP+ as acceptor	Unkonw
R00351	Acetyl‐CoA:oxaloacetate C‐acetyltransferase (thioester‐hydrolysing)	CoA + H^+^ + Citrate ← H_2_O + Acetyl‐CoA + Oxaloacetate	EC 2.3.3.1_;L1_Transferase reactions;L2_Acyltransferases;L3_Acyl groups converted	Unkonw
R00405	Succinate:CoA ligase (ADP‐forming)	ATP + CoA + Succinate ← ADP + Phosphate + Succinyl‐CoA	EC 6.2.1.5_;L1_Ligase reactions;L2_Forming carbon‐sulfur bonds;L3_Acid‐thiol ligases	Increases the sour salty and bitter taste (Torres‐Guardado et al. 2022)
R00703	(S)‐Lactate:NAD+ oxidoreductase	NAD + L‐Lactate ↔ NADH + Pyruvate + H^+^	EC 1.1.1.27_;L1_Oxidoreductase reactions;L2_Acting on the CH‐OH group of donors;L3_With NAD+ or NADP+ as acceptor	Unknown
R00709	Isocitrate:NAD+ oxidoreductase (decarboxylating)	NAD + Isocitrate → NADH + CO_2_ + 2‐Oxoglutarate	EC 1.1.1.41_;L1_Oxidoreductase reactions;L2_Acting on the CH‐OH group of donors;L3_With NAD+ or NADP+ as acceptor	Influence the structure and balance of wine
R01082	(S)‐malate hydro‐lyase (fumarate‐forming)	L‐Malate ← H_2_O + Fumarate	EC 4.2.1.2_;L1_Lyase reactions;L2_Carbon‐oxygen lyases;L3_Hydro‐lyases	Unkonw
R01324	Citrate hydroxymutase	Citrate → Isocitrate	EC 4.2.1.3_;L1_Lyase reactions;L2_Carbon‐oxygen lyases;L3_Hydro‐lyases	Increase aroma complexity
R01513	3‐Phospho‐D‐glycerate:NAD+ 2‐oxidoreductase	NAD + 3‐Phosphoglycerate ↔ NADH + H^+^ + 3‐Phosphonooxypyruvate	EC 1.1.1.95_;L1_Oxidoreductase reactions;L2_Acting on the CH‐OH group of donors;L3_With NAD+ or NADP+ as acceptor	Carbohydrate metabolism
R04125	S‐aminomethyldihydrolipoylprotein:(6S)‐tetrahydrofolate aminomethyltransferase (ammonia‐forming)	Tetrahydrofolate + S‐Aminomethyldihydrolipoylprotein → NH_3_ + 5–10‐Methylenetetrahydrofolate + Dihydrolipolprotein	EC 2.1.2.10_;L1_Transferase reactions;L2_Transferring one‐carbon groups;L3_Hydroxymethyl‐, formyl‐ and related transferases	Unkonw
R08549	2‐Oxoglutarate dehydrogenase complex	NAD + CoA + 2‐Oxoglutarate → NADH + CO_2_ + Succinyl‐CoA	EC 1.2.1.105_;L1_Oxidoreductase reactions;L2_Acting on the aldehyde or oxo group of donors;L3_With NAD+ or NADP+ as acceptor	Antioxidative defence

*Note:* It includes key information such as the Reaction ID, the catalysing enzyme, the complete reaction representation and the Enzyme Commission (EC) number, providing a comprehensive classification of the enzyme involved (level 1, level 2 and level 3). The numerical values within the brackets denote the stoichiometry of the reaction.

In summary, *L. hilgardii* showcases a robust metabolic potential with positive implications for winemaking. Using genome‐scale models and simulations in rich and synthetic grape juice media in post‐alcoholic fermentation, the study reveals key differences from *O. oeni* , emphasising *L. hilgardii* 's unique enzymes impacting carbohydrate metabolism. Notably, enzymes like alpha‐D‐glucose 1‐phosphate 1,6‐phosphomutase and 3‐phospho‐D‐glycerate:NAD+ 2‐oxidoreductase play crucial roles in citric acid cycle modulation, citrate metabolism and enhancing wine aroma complexity. The presence of the 2‐oxoglutarate dehydrogenase complex suggests influences on antioxidative defence and sulfur compound binding, with implications for wine aging. Exclusive enzymes like D‐alanine:D‐alanine ligase hint at bacterial resilience during MLF. Under glucose‐depleted conditions, *L. hilgardii* exhibits unique reactions related to malolactic fermentation, highlighting potential novel pathways. Although aminomethyltransferase isn’t linked to winemaking context in the literature, its presence in *L. hilgardii* sparks intriguing possibilities. In conclusion, *L. hilgardii* 's metabolic versatility might positively impacts aroma, organic acids, antioxidative defence and resilience in challenging fermentation conditions during wine production.

The versatility of *L. hilgardii* compared to *O. oeni* can influence the inoculation method. For this reason, we tested the reactions applied by *L. hilgardii* in pre‐alcoholic fermentative conditions using a genome‐scale model. We compared co‐inoculation with *Saccharomyces cerevisiae* EC1118, the most commonly used yeast strain for alcoholic fermentation, against co‐inoculation with *Oenococcus oeni* . Our findings indicate that several reactions occur exclusively with the co‐inoculation of *L. hilgardii* and *S. cerevisiae* . These reactions are listed in Table 4.

**TABLE 4:** The table shows the potential reactions simulated during alcoholic fermentation when *L. hilgardii* is co‐inoculated with *S. cerevisiae* EC1118, excluding the reactions common to the co‐inoculation of *O. oeni* and *S. cerevisiae* .

Reaction id	Enzyme	Reaction	EC
R01150	D‐alanine:D‐alanine ligase (ADP‐forming)	ATP + (2) D‐Alanine → ADP + Phosphate + H+ + Ala‐Ala	EC 6.3.2.4;L1_Ligases;L2_Forming carbon‐nitrogen bonds;L3_Acid—amino‐acid ligases (peptide synthases)
R00332	ATP:GMP phosphotransferase	ATP + H+ + GMP → ADP + GDP	EC 2.7.4.8;L1_Transferases;L2_Transferring phosphorus‐containing groups;L3_Phosphotransferases with a phosphate group as acceptor
R00192	S‐Adenosyl‐L‐homocysteine hydrolase	H2O + S‐Adenosyl‐homocysteine → Homocysteine + Adenosine	EC 3.13.1.9;L1_Hydrolases;L2_Acting on carbon‐sulfur bonds;L3_Acting on carbon‐sulfur bonds
R00619	ATP:thiamine diphosphotransferase	ATP + Thiamin → AMP + TPP + H+	EC 2.7.6.2;L1_Transferases;L2_Transferring phosphorus‐containing groups;L3_Diphosphotransferases
R00200	ATP:pyruvate 2‐O‐phosphotransferase	ATP + Pyruvate → ADP + Phosphoenolpyruvate + H+	EC 2.7.1.40;L1_Transferases;L2_Transferring phosphorus‐containing groups;L3_Phosphotransferases with an alcohol group as acceptor
R02821	Trimethylaminoacetate:L‐homosysteine S‐methyltransferase	Homocysteine + BET → L‐Methionine + Dimethylglycine	EC 2.1.1.9;L1_Transferases;L2_Transferring one‐carbon groups:L3_Methyltransferases
R00330	ATP:GDP phosphotransferase	ATP + GDP → ADP + GTP	EC 2.7.4.1;L1_Transferases;L2_Transferring phosphorus‐containing groups;L3_Phosphotransferases with a phosphate group as acceptor
R01856	dGTP triphosphohydrolase	H2O + dGTP ← Deoxyguanosine + Triphosphate	EC 3.1.5.1;L1_Hydrolases;L2_Acting on ester bonds;L 3_Triphosphoric‐monoester hydrolases
R00362	Citrate oxaloacetate‐lyase (forming acetate from the pro‐S carboxymethyl group of citrate)	Citrate ← Acetate + Oxaloacetate	EC 4.1.3.6;L1_Lyases;L2_Carbon‐carbon lyases;L3_Oxo‐acid‐lyases
R00158	ATP:UMP phosphotransferase	ATP + H+ + UMP → ADP + UDP	EC 2.7.4.1;L1_Transferases;L2_Transferring phosphorus‐containing groups;L3_Phosphotransferases with a phosphate group as acceptor

*Note:* It includes key information such as the Reaction ID, the catalysing enzyme, the complete reaction representation, and the Enzyme Commission (EC) number, providing a comprehensive classification of the enzyme involved (level 1, level 2 and level 3). The numerical values within the brackets denote the stoichiometry of the reaction.

The differential metabolic responses observed from the co‐inoculation of *L. hilgardii* and *S. cerevisiae* involved the expression of six transferases, two ligases and two hydrolases. Some of these reactions did not occur in sequential fermentation, suggesting their potential oenological importance in co‐inoculation. For instance, the expression of S‐adenosyl‐L‐homocysteine hydrolase in yeast is subject to the general transcriptional control of phospholipid synthesis. Significant changes in cellular lipid composition upon the depletion of the gene responsible for S‐adenosyl‐L‐homocysteine hydrolase support the notion of a tight interaction between lipid metabolism and this enzyme's function (Tehlivets et al. 2004).

ATP diphosphotransferase catalyses the conversion of thiamine to thiamine diphosphate (also known as thiamine pyrophosphate or TPP), a biologically active form of thiamine. TPP is an essential cofactor for several enzymes, such as pyruvate dehydrogenase, α‐ketoglutarate dehydrogenase and transketolase, which are crucial for central metabolic pathways including the citric acid cycle and the pentose phosphate pathway (de Jong et al. 2004).

Trimethylaminoacetate S‐methyltransferase plays a significant role in oenology by influencing the metabolism of sulfur‐containing amino acids. This contributes to aroma and flavour development, supports yeast health and fermentation efficiency, and ultimately affects the stability and quality of wine.

Citrate oxaloacetate‐lyase helps modulate acidity by breaking down citric acid. The reaction catalysed by citrate lyase leads to the production of oxaloacetic acid, which is converted by oxaloacetate decarboxylase into pyruvate. Pyruvate is primarily reduced to lactate in the presence of NADH; however, some pyruvate is converted by acetolactate decarboxylase to acetolactic acid, which, following decarboxylation, gives rise to acetoin and 2,3‐butanediol. The chemical oxidation of acetoin yields diacetyl. The precursor of diacetyl (and acetoin), α‐acetolactate, is also an intermediate in the biosynthesis of the amino acids valine and leucine. The degradation of citric acid by LABs consequently leads to an increase in volatile acidity in wine (Belda et al. 2017).

### 3.8 Comparison Between the Reference Genome of *O. oeni* and *L. hilgardii*

This study involved the analysis of two reference genomes for each species: AWRIB429 served as the reference genome for *O. oeni* , while FLUB represented the reference genome for *L. hilgardii* . To enhance our comprehension, an additional observational analysis was performed to compare pathways using the RAST platform. In this case, exclusive reliance on genomes derived from core genes proved impractical. This alternative approach provided additional insights, particularly in elucidating the quantity of genes associated with specific pathways. The information about the two genomes was detailed in the Table 5.

**TABLE 5:** Organisms overview of the two reference genomes: *O. oeni* AWRIB429 and *L. higlardii* FLUB.

Information	AWRIB429	FLUB
Size	1′892′074	3′190′226
GC content	38.1	40.1
N50	1′870′168	3′071′102
L50	1	1
Number of contigs	2	6
Number of Subsystems	196	221
Number of Coding Sequences	2′067	3′136
Number of RNAs	49	76
Subsystem Coverage	26%	22%

**FIGURE 6:**
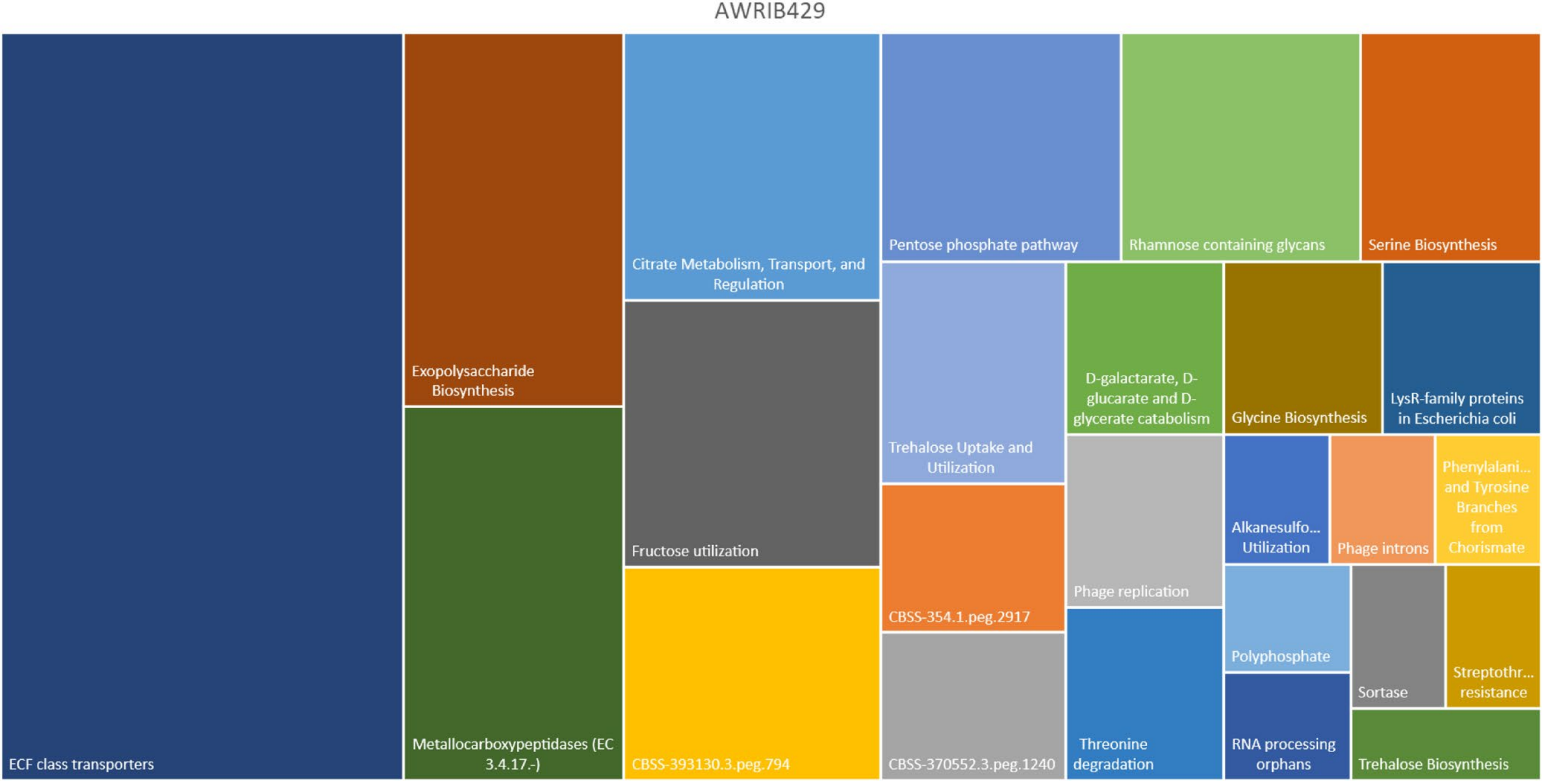
A visually informative treemap illustrates the proportional distribution within hierarchical levels, representing potential pathways identified in the *Oenococcus oeni* AWRIB429 strain. Within this treemap, rectangles are employed to depict the diverse hierarchy levels, offering a representation of the proportion of genes associated with specific pathways.

Relatively to these data, interesting diversities were found between the two strains from an enzymatic perspective. AWRIB429 possesses various classes of enzymes (Figure 6), some directly or indirectly involved in the winemaking process.

Among these is the catabolic pathway of D‐galactarate, D‐glucarate and D‐glycerate catabolism, leading to the catabolism of D‐galacturonic acid, a major component of pectin (Protzko et al. 2019). Grape juice is naturally rich in polysaccharides like pectin, cellulose, hemicellulose and other substances, mainly derived from grape cell walls and middle lamellae. The high pectin content, in particular, leads to the formation of a colloidal structure that can complicate must fermentation (Sandri et al. 2011). Bacteria capable of degrading pectin are used during secondary fermentation to avoid these colloidal structures, which can lower the quality of the final product.

The production of phenylalanine and tyrosine branches from chorismate is another pathway found in the AWRIB429 strain, involving the conversion of chorismate by chorismate mutase into prephenate, followed by the conversion of prephenate into phenylalanine and tyrosine. In winemaking, these aromatic amino acids have oenological significance as they act as precursors for the formation of numerous aromatic compounds contributing to the wine's flavour and aroma.

Threonine degradation is another enzyme that can influence the final outcome of winemaking, found in the *O. oeni* strain. In the context of wine, amino acids, including threonine (the fifth most abundant amino acid in must), play a significant role in various biochemical transformations during the winemaking process, catalysed by specific enzymes belonging to the pathway dedicated to threonine degradation. According to several studies, amino acids constitute a potential source of the furanone compound in wines. The first description of sotolon, a chiral furanone responsible for imparting distinct flavour characteristics, formation via the degradation product of threonine, was historically provided by Sulser (Sulser et al. 1967). Sotolon is known for causing a premature aging flavour in dry white wines (Pons et al. 2010), imparting a nutty, toasted aroma to wine (Cutzach et al. 1998).

**FIGURE 7:**
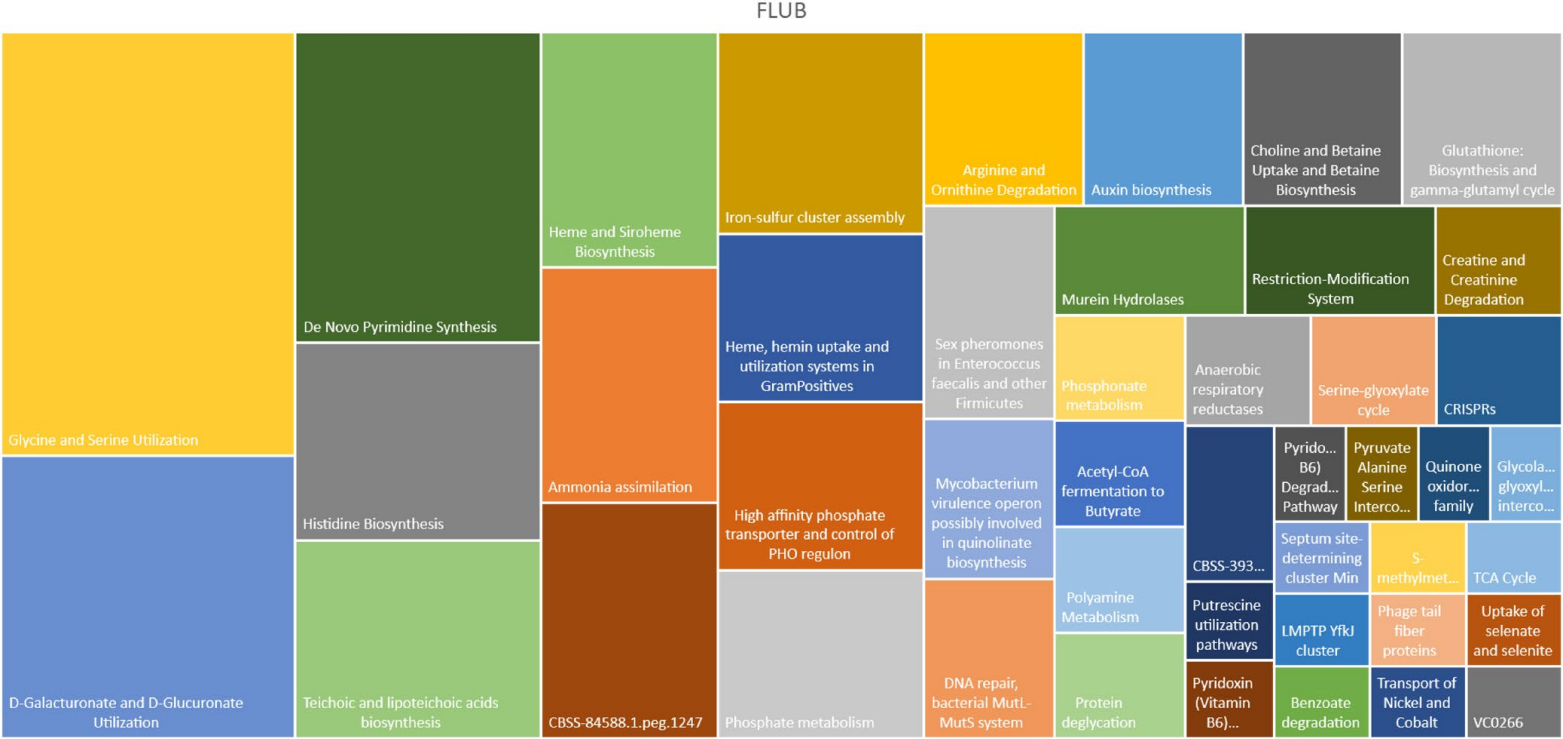
A visually informative treemap illustrates the proportional distribution within hierarchical levels, representing potential pathways identified in the *Lentilactobacillus hilgardii* FLUB strain. Within this treemap, rectangles are employed to depict the diverse hierarchy levels, offering a representation of the proportion of genes associated with specific pathways.

Concerning the enzymatic classes found in *L. hilgardii* FLUB (Figure 7), the ammonia assimilation class was identified. Assimilable nitrogen is the combination of free amino nitrogen (FAN), ammonia (NH_3_), and ammonium (NH_4_^+^) available during fermentation. Nitrogen is the most crucial nutrient for ensuring a successful fermentation, preventing premature termination and avoiding the development of undesirable odours and wine‐related defects (Santamaría et al. 2020). The arginine and ornithine degradation pathways hold particular significance for wine safety, presenting both positive and negative implications. On the downside, LAB can leverage this pathway, particularly through the arginine deiminase (ADI) pathway, to degrade arginine in wine. This degradation leads to the production of citrulline and carbamoyl phosphate, serving as precursors for ethyl carbamate—a substance of concern in wine production due to its potential carcinogenic effects.

Conversely, the pathway introduces a positive aspect by involving ornithine, a common intermediate in the degradation of arginine and citrulline. Specifically, the ornithine degradation refers to the reaction catalysed by L‐ornithine:2‐oxo‐acid aminotransferase: L‐Ornithine +2‐Oxoglutarate < = > L‐Glutamate 5‐semialdehyde + L‐Glutamate. Specific variants of LAB present in wine can convert citrulline into ornithine and ammonia, effectively reducing the precursor for ethyl carbamate. This mechanism acts as a positive feedback loop, mitigating the formation of ethyl carbamate and contributing to overall wine safety. In conclusion, while the arginine and ornithine degradation pathway poses the potential risk of ethyl carbamate production, the concurrent reduction of its precursor demonstrates a dual impact on wine safety. The intricate interplay of these processes underscores the necessity for meticulous consideration and monitoring in winemaking practices to ensure both the quality and safety of the final product.

Another enzymatic class found exclusively in the *L. hilgardii* FLUB strain involves choline and betaine uptake and betaine biosynthesis. Recent studies have demonstrated that betaine supplementation can enhance the performance of microbial strains used for the fermentation of various compounds, acting as a stress protector or a methyl donor for the biosynthesis of structurally complex compounds (Zou et al. 2016). Compounds like betaine have been proven to protect LAB during freeze‐drying. This capability could also be interesting for biomass production, as it may improve the viability and robustness of microbial cultures under various stress conditions (Louesdon et al. 2014). Additionally, the pathways found with oenological significance for the *L. hilgardii* strain are the glutathione biosynthesis and gamma‐glutamyl cycle, by slightly increasing the expression of the gsh1 gene encoded for gamma‐glutamylcysteine synthetase involved in glutathione biosynthesis during fermentation. Glutathione is the most abundant non‐protein thiol commonly found in yeasts and plays an important role as an antioxidant in wine. The application of selected bacterial strains that accumulate glutathione can improve the sensory quality and stability of wine (Lemos Junior et al. 2021). Another class found uniquely in FLUB is D‐galacturonate and D‐glucuronate utilisation, degrading D‐galacturonate and D‐glucuronate, key constituents of pectin. Pectin is a ubiquitous monomer in plant biomass, as mentioned earlier, and it can pose an issue for wine turbidity (Romat and Renouf 2020).

Furthermore, a pathway for the utilisation of glycine and serine was also observed. A study found that glycine, along with other amino acids such as tyrosine, leucine and lysine, was positively correlated with the production of fusel alcohols and acetate esters. These compounds contribute to the aroma and taste of the wine. The study suggested that the overall aroma production is a function of nitrogen utilisation, which includes the use of amino acids such as glycine and serine (Fairbairn et al. 2017).

The biosynthesis of histidine was also found uniquely in *L. hilgardii* FLUB. Histidine is one of the most studied amino acids of wines due to histamine toxicity in humans, a biogenic amine derived from histidine by enzymatic decarboxylation (López‐Rituerto et al. 2013). Winemakers must carefully monitor and manage the microbial composition during MLF to minimise the risk of histamine production and ensure the safety and quality of their wines.

In the last years, it has been reported the ability of *L. plantarum* strains to reduce the tyramine and putrescine content in wine, while multicopper oxidase enzymes responsible for the degradation of histamine, tyramine and putrescine have been isolated and purified from *L. plantarum* and *Pediococcus acidilactici* (Capozzi et al. 2012; Callejón et al. 2014). Interestingly, pathways for the utilisation of putrescine, the most abundant biogenic amine in wine (Mangani et al. 2005), were also found in *L. hilgardii* FLUB, suggesting potential application in wine due to the ability to degrade this biogenic amine.

Interconversions of pyruvate, alanine and serine were noted in the genome of *L. hilgardii* FLUB. In the context of wine, these amino acids play a significant role. They are involved in many biochemical transformations during the winemaking process, catalysed by specific enzymes. These compounds participate in the formation of other aminoacids, which also have fundamental functions in the sensory quality of wine. Therefore, the interconversions of pyruvate, alanine and serine can influence the fermentation process in wine production, affecting the final quality of the product (Scutarașu et al. 2022).

Furthermore, an enzyme involved in the production of S‐methylmethionine was also found, that could be of peculiar interest in winemaking. These non‐volatile and odourless constituents are susceptible to transforming into volatile aromatic compounds during the biotechnological sequence of winemaking, from the disorganisation of grape cells during harvesting to the maturation of wine during storage (Jiménez‐Lorenzo et al. 2022).

Pathways for the absorption of selenate and selenite were also identified, playing a crucial role in the wine fermentation process. This can lead to an increase in phytochemical profiles, flavour, quality and antioxidant capacity of the wine (Assunção et al. 2015; Congcong et al. 2023).

It is important to acknowledge some caveats related to the bioinformatic modelling approach used in this study. Genome‐scale metabolic models (GEMs) and in silico co‐inoculation simulations, as implemented through the KBase/ModelSEED platform, provide valuable insights into metabolic capabilities but rely on automated genome annotation and reaction gap‐filling. These processes may introduce biases, particularly when dealing with species for which curated models are not yet available, such as *L. hilgardii* . Moreover, GEM predictions do not account for complex regulatory networks, environmental stress responses, or metabolite transport limitations that can affect in vivo performance. Nonetheless, several studies have demonstrated the reliability of KBase‐based models in predicting fermentation dynamics and metabolic fluxes in lactic acid bacteria (Edirisinghe et al. 2016; Kristjansdottir et al. 2019), supporting the robustness of the approach while highlighting the need for complementary experimental validation, as carried out in this work.

This study is subject to a notable limitation as it addresses the potential genetic capabilities of *L. hilgardii* in comparison to *O. oeni* . However, it is crucial to acknowledge that this comparison lacks, in part, empirical validation in wet lab experiments.

In summary, *L. hilgardii* FLUB displays a positive and diverse enzymatic profile, impacting various aspects of winemaking. Exclusive pathways like choline and betaine uptake and betaine biosynthesis offer potential benefits for microbial strain performance. Despite the potential risk of ethyl carbamate production, the arginine and ornithine degradation pathway contributes to wine safety. Unique classes such as glutathione biosynthesis and gamma‐glutamyl cycle enhance antioxidant properties. Specific pathways address turbidity issues and contribute to aroma, taste and quality improvement. Interconversions and production of S‐methylmethionine influence fermentation and sensory quality. Absorption of selenate and selenite contributes to the phytochemical profile and antioxidant capacity. While empirical validation is needed, *L. hilgardii* 's genetic capabilities suggest it as a valuable contributor to aroma development, and overall wine quality.

## 4 Conclusion

This study provides the first integrated genomic, metabolic and experimental assessment of *Lentilactobacillus Hilgardii* as a potential malolactic starter in winemaking. Our results show That *L. hilgardii* shares key MLF‐related traits with *Oenococcus oeni* while possessing unique genomic features related to stress tolerance and aroma modulation. Notably, *L. hilgardii* efficiently completed malolactic fermentation at low temperature, outperforming *O. oeni* under the same conditions. These findings highlight *L. hilgardii* as a promising candidate to Expand the biodiversity of malolactic starters and improve fermentation reliability, particularly in cool‐climate winemaking.

## Author Contributions

G.G., G.M. conceived and designed the project. E.V.Y., G.M. performed the analyses concerning the malolactic enzyme molecular docking. G.G. performed all the other analyses, and wrote the initial draft of the manuscript. N.M. performed all the experimental part of the study. All the authors contributed to refining and expanding the manuscript through their valuable input, suggestions, and assistance in writing.

## Conflicts of Interest

The authors declare no conflicts of interest.

## Data Availability

The data that support the findings of this study are publicly available in the NCBI database.
