# Correction to ‘Unveiling the Potential of *Lentilactobacillus hilgardii* in Malolactic Fermentation: Comparative Genomics and Fermentation Dynamics’

**DOI:** 10.1111/1751-7915.70402

**Published:** 2026-06-18

Mantegazza, G. , N. Mangieri , E. V. Yazdi , P. Russo , D. Mora , and G. Gargari . 2025. “Unveiling the Potential of *Lentilactobacillus hilgardii* in Malolactic Fermentation: Comparative Genomics and Fermentation Dynamics.” Microbial Biotechnology 18, no. 12: e70259. 10.1111/1751-7915.70259.41319147 PMC12665152

In the original manuscript, Figure 5 was incorrect; however, the figure legend and the corresponding text were accurate. The correct version of Figure 5 is provided below, along with the incorrect version for reference.

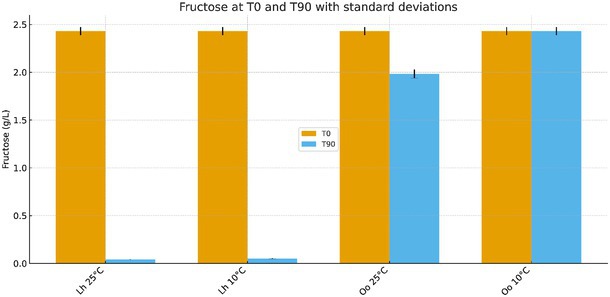

FIGURE 5 **CORRECT** | Fructose concentration (g/L) at Day 0 and Day 90 during fermentation with *Lentilactobacillus hilgardii* and *Oenococcus oeni* at 25°C and 10°C. Data are presented as mean ± standard deviation (*n* = 3). *L. hilgardii* exhibited a markedly higher capacity for fructose consumption under both thermal conditions, whereas *O. oeni* showed limited or no utilisation, particularly at low temperature.

The incorrect figure present now in the manuscript is shown below:

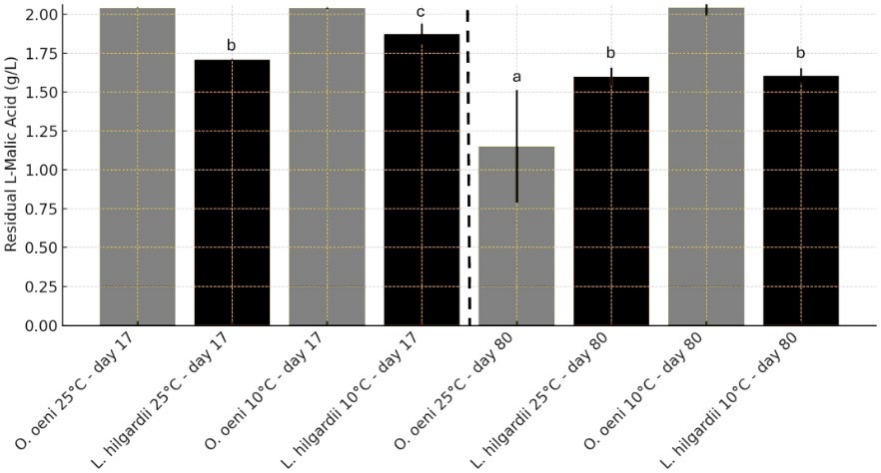

FIGURE 5 **INCORRECT**.

We apologize for this error.

